# STRA6 Polymorphisms Are Associated With EGFR Mutations in Locally-Advanced and Metastatic Non-Small Cell Lung Cancer Patients

**DOI:** 10.3389/fonc.2020.579561

**Published:** 2020-11-24

**Authors:** Saé Muñiz-Hernández, Jesús Bernardino Velázquez-Fernández, José Díaz-Chávez, Omar Mondragón-Fonseca, Yerye Mayén-Lobo, Alberto Ortega, Marisol López-López, Oscar Arrieta

**Affiliations:** ^1^ Laboratorio de Oncología Experimental, Subdirección de Investigación Básica, Instituto Nacional de Cancerología, Ciudad de México, Mexico; ^2^ Unidad de Tecnología Ambiental, Centro de Investigación y Asistencia en Tecnología del Estado de Jalisco, Jalisco, Mexico; ^3^ Laboratorio de Carcinogénesis, Dirección de Investigación, Instituto Nacional de Cancerología, Ciudad de México, Mexico; ^4^ Laboratorio de Genética Molecular, Departamento de Sistemas Biológicos, Universidad Autónoma Metropolitana-Xochimilco, Ciudad de México, Mexico; ^5^ Unidad de Oncología Torácica, Instituto Nacional de Cancerología, Ciudad de México, Mexico

**Keywords:** non-small cell lung cancer, single nucleotide polymorphisms, stimulated by retinoic acid 6 (STRA6), genotype, retinol pathways

## Abstract

Retinol plays a significant role in several physiological processes through their nuclear receptors, whose expression depends on retinol cytoplasmic concentration. Loss of expression of nuclear receptors and low retinol levels have been correlated with lung cancer development. Stimulated by retinoic acid 6 (STRA6) is the only described cell membrane receptor for retinol uptake. Some chronic diseases have been linked with specific polymorphisms in STRA6. This study aimed to evaluate four STRA6 single nucleotide polymorphisms (SNPs) (rs4886578, rs736118, rs351224, and rs97445) among 196 patients with locally-advanced and metastatic non-small cell lung cancer (NSCLC) patients. Genotyping, through a validated SNP assay and determined using real time-PCR, was correlated with clinical features and outcomes. NSCLC patients with a TT SNP rs4886578 and rs736118 genotype were more likely to be >60 years, non-smokers, and harboring EGFR mutations. Patients with a TT genotype compared with a CC/CT SNP rs974456 genotype had a median progression-free survival (PFS) of 3.2 vs. 4.8 months, p = 0.044, under a platinum-based regimen in the first-line. Furthermore, patients with a TT rs351224 genotype showed a prolonged overall survival (OS), 47.5 months vs. 32.0 months, p = 0.156. This study showed a correlation between clinical characteristics, such as age, non-smoking history, and EGFR mutational status and oncological outcomes depending on STRA6 SNPs. The STRA6 TT genotype SNP rs4886578 and rs736118 might be potential biomarkers in locally-advanced and metastatic NSCLC patients.

## Introduction

Lung cancer is the leading cause of cancer-related deaths worldwide and is currently responsible for 1.8 million deaths per year; moreover, it will soon represent around 30% of all cancer-related deaths (1). Non-small cell lung cancer (NSCLC) accounts for approximately 85% of all lung cancers. In Mexico, this tumor subtype is the leading cause of cancer deaths, with about 7,000 deaths per year (2, 3), and almost 90% of them were diagnosed in advanced stages (4–6).

Retinoids are a family of signaling molecules related to vitamin A (retinol), and play a significant role in several cellular processes, such as embryogenesis, cell proliferation, differentiation, and apoptosis (7). Moreover, retinoids have been demonstrated to stop carcinogenesis in breast, lung, prostate, bladder, and lung cancer (7–10). Retinoid effects occurred due to the activation of the nuclear retinoic acid receptors (RARs) and retinoid X receptors (RXRs), responsible for developing pulmonary epithelium and lung cancer pathogenesis (11, 12). Evidence indicates NSCLC patients overexpress RXRα and RARγ; conversely, RARβ suppression exists, suggesting an imbalance in the receptors’ expression that could play an essential role in cancer genesis (12). Preclinical and clinical studies demonstrated that retinoids might reverse premalignant epithelial lesions and preventing secondary neoplasms.

Nuclear retinol receptor expression depends on cytoplasm concentrations, and the plasma retinol-binding protein (RBP) is the primary transporter for retinol in blood. Retinol enters the cells from the RBP *via* a cell-surface receptor, named STRA6 (Stimulated by Retinoic Acid 6) (13, 14). The exogenous addition of retinoic acid has increased RAR levels in preclinical models (15). Furthermore, natural or synthetic retinoids added to paclitaxel and cisplatin-based chemotherapy has improved the median overall survival (OS) in advanced NSCLC patients (9, 16).

STRA6 is a cell-surface membrane protein located predominantly in blood-organ barriers and expressed during embryonic development and adult stages (17, 18). It belongs to a large group of retinoic-acid-stimulated genes encoding transmembrane proteins whose function remains unknown (19). STRA6 gene is contained in the chromosome 15q24.1 region and made up of 20 exons and 19 introns encoding for a 667 residues protein. However, until now, the topology and transportation mechanisms of the human STRA6 gene remains unknown. STRA6 is overexpressed in the colon, breast, and gastric tumor tissue, suggesting a tumorigenesis role (18, 20, 21). Recently, an *in-vitro* study showed that STRA6 is down-regulated by miR-873 in gastric cancer cells; (22) yet, scarce information about its role in lung cancer pathogenesis is available.

STRA6 mutations and single nucleotide polymorphisms (SNPs) alter its function and cellular topography, mainly in the RBP-binding domain (23). The Matthew-Wood syndrome, a severe pathological phenotype characterized by malformations in multiple human organs, including the eye, brain, heart, and lung, has been associated with some STRA6 mutations (24, 25). Prior reports have shown an association of type 2 diabetes mellitus (DM2) in Chinese and Indian populations with 3 SNPs (rs736118, rs974456, and rs4886578); however, the allelic variation expression varies within these populations (26, 27). Limited information concerning *STRA6* polymorphisms in the Mexican population has been documented. Furthermore, information regarding *STRA6* genetic variability and its relationship with lung cancer remains unexplored. This study aims to analyze four *STRA6* SNPs (rs4886578, rs736118, rs351224, rs974456) and their association with clinical features, PFS, and OS in locally-advanced and metastatic NSCLC patients.

## Materials and Methods

### Study Design

One hundred ninety-six patients with confirmed NSCLC patients treated in the Thoracic Oncology Unit at the Instituto Nacional de Cancerología (INCan) in Mexico City from January 2015 to December 2016, were eligible. Inclusion criteria were patients with locally-advanced and metastatic NSCLC (stage IIIA–IVC), according to the American Joint Committee on Cancer (AJCC) 8th edition cancer staging, malignant pleural effusion carriers, Eastern Cooperative Oncology Group Scale Performance Status (ECOG PS) 0–2. The present research was approved by the Institutional Review Board and Ethics Committee of the Instituto Nacional de Cancerología of Mexico, [INCAN (015/026/IBI) (CEI/954/15)]. All patients provided written informed consent before enrollment and any study-specific procedures.

### Clinical Data Collection, Treatment Regimen, and Patient Follow-Up

All relevant clinical features from eligible patients were obtained from electronic medical records. Chosen therapy was at consideration of treating physicians in agreement with international guidelines (4). Most of the patients received platinum-based chemotherapy as first-line treatment in combination with paclitaxel or pemetrexed. After progression to first-line chemotherapy, EGFR mutated patients received EGFR- tyrosine kinase inhibitors (EGFR-TKIs) as further therapy lines.

### *STRA6* Genotyping

A peripheral blood sample was obtained (4 ml) in every patient to evaluate genotyping. Genomic DNA was obtained using the Wizard*^®^* Genomic DNA Purification kit (Promega*^®^*, Wisconsin, USA). Real-time PCR (RT–PCR) was used for genotyping the different allelic variants. The SNPs NC_000015.10:g.74180694C>T (rs4886578), NC_000015.10:g.74181398C>T (rs736118), NC_000015.10:g.74194695T>A (rs351224) and NC_000015.10:g.74194103C>T (rs974456), were genotyped with commercially available TaqMan validated SNP assays (C_32357728_10, C_966873_10C, C_3152256_10 and C_3152257_20, respectively) from Applied Biosystems (Applied Biosystems, Massachusetts, USA). Amplification was performed following manufacturer instructions using a Light Cycler 480 RT-PCR instrument (Roche, Indiana, USA). The conditions of denaturation, alignment, and amplification were 94°C - 30 s, 55°C - 1 min, and 72 °C - 30 s for a total of 40 cycles. An allelic discrimination plot was used to identify individual genotypes by mean the light cycler software v 1.5.0 sp4 (Roche, Indiana, USA).

Subsequently and independently, each allelic variance was confirmed by direct sequencing in five percent of the samples and was amplificated based on its high integrity and purity. Primers were designed using Primer Express Software v.3 (Thermo Fisher Scientific Inc. Massachusetts, USA): rs4886578 (5’-CCTCCCTGGCCCTCAGA-3’ and 3’-TCGCATCCAGCCATGACA-5’); rs736118 (5’-CATCCAGACATCCCCTAACACA-3’ and 3’-CGGCCTGCCTCAGCTTT-5’); rs351224 (5’-CACCCTGAGGGACTGGTGTT-3’ and 3’-GAGCCAGACAACCTGAGTGT GA-5’); rs974456 (5’-GCTGCACAGGGTACCAAAGG-3’ and 5’-CGGGAACACAGAAAC AGAAAGAG-3’). The amplification conditions have been established by aligning gradient temperature on an end-point thermocycler. Purified amplicons were sequenced with Big Dye Terminator v3.1 Cycle Sequencing Kit (Thermo Fisher Scientific Inc. Massachusetts, USA) and separated in a genetic analyzer AB3130 (Applied Biosystems, Massachusetts, USA). All analyzed sequences were evaluated with a Sequencing Analysis software v5.3 (Thermo Fisher Scientific Inc. Massachusetts, USA), and every allelic variant was confirmed by reference sequencing (NC_000015.10); the product of an independent PCR on both strands and verified in different public databases. Genotype frequencies were tested for Hardy-Weinberg equilibrium by using the Haploview software v4.2 (Mark Daly Laboratory, Massachusetts Institute of Technology/Harvard Broad Institute, Cambridge, MA, USA) (28).

Linkage disequilibrium (LD) and haplotype analysis were performed using the Haploview software v 4.2 (28) (MIT/Harvard Broad Institute, Cambridge, MA, USA). Haplotype block was defined based on Gabriel et al., 2002 description (D’ > 0.9; minimum allelic frequency >5%) (29).

### Statistical Analysis

For descriptive purposes, continuous variables were summarized as arithmetic means and standard deviations (SD), while categorical variables were presented as frequencies and proportions. The student t-test was employed for assessing differences among continuous variables. Chi-squared or Fisher’s exact tests were used to assess the significant differences among categorical variables. For the survival analysis, variables were dichotomized. Progression-free survival (PFS) was defined as the time from the date of initiating treatment to radiologic or clinical disease progression or death from any cause, whichever occurred first. Patients without progression or death, including those who discontinued treatment due to unacceptable toxicity or were lost to follow-up, were censored at the date of last disease assessment or last contact. In contrast, overall survival (OS) was defined as the time from diagnosis until death or loss to follow-up. PFS and OS were estimated using the Kaplan-Meier method; log-rank tests were employed to set comparisons among subgroups. Adjustment for potential confounders was performed using a multivariate Cox regression model, and hazard ratios (HR) were calculated along with their corresponding 95% confidence intervals (CI) as a measure of association. For each clinical factor, we performed a univariate analysis followed by multivariable Cox Regression analysis. The inclusion criteria for variables in multivariable analyses was a p >0.10. A p-value <0.05 was considered significant based on a two-sided test. All statistical analyses were performed using the SPSS software package, v. 15 (SPSS Inc, Chicago, IL).

## Results

### Study Population

The median age at diagnosis was 61 (± 12.5) years; most of the patients were female (56.1%), without wood-smoke exposure (55.1%), and half of them were current/former smokers. Adenocarcinoma subtype was the most common subtype in 85.7%, and 40% of the total cohort (73/196) had a positive EGFR mutational status. All baseline characteristics are summarized in **Table 1**. Patients received a platinum-based chemotherapy regimen in 84.2% (165/196) as first-line treatment, and 86 (43.8%) of the cases harboring an EGFR mutation received as second or third line an EGFR-TKI. Gefitinib was the most commonly used TKI (17.9%), followed by afatinib (16.8%) and erlotinib (7.7%) (**Table S1**).

**Table 1:** General characteristics of patients.

	N = 196% (n/N)
**Gender**	
Female	56.1 (110/196)
Male	43.9 (86/196)
**Age**	
Mean (± SD)	61.0 (± 12.5)
≤ 60 yrs	45.4 (89/196)
> 60 yrs	54.6 (107/196)
**Tobacco history**	
Smoker	50 (98/196)
Non-smoker	50 (98/196)
**Passive tobacco exposure**	
Yes	10.7 (21/196)
No	89.3 (175/196)
**Asbestos exposure**	
Yes	15.8 (31/196)
No	84.2 (165/196)
**Wood-smoke exposure**	
Yes	44.9 (88/196)
No	55.1 (108/196)
**ECOG**	
0–1	71.4 (140/196)
2–3	28.6 (56/196)
**Disease Stage**	
III	11.7 (23/196)
IV	88.3 (173/196)
**Diabetes Diagnosis**	
Yes	14.8 (29/196)
No	84.7 (166/196)
**Glycaemia**	
<120	77.6 (152/196)
≥120	22.4 (44/196)
**Histology**	
Adenocarcinoma	85.7 (168/196)
Other	14.3 (28/196)
**EGFR status**	
Wilt-type	62.8 (123/196)
Mutated	37.2 (73/196)
**EGFR mutation**	
Exon 19 (deletion)	26.5 (52/73)
Exon 20 (T790M)	0.5 (1/73)
Exon 21 (L858R)	10.2 (20/73)

SD, standard deviation; ECOG, European Cooperative Eastern Group Classification.

A prior diagnosis of diabetes mellitus (DM) was self-reported by 14.8% (29/196) of the participants (**Table 1**). However, serological fasting glucose levels were impaired (100-125 mg/dL) in sixty individuals (35.9%, 60/167). Moreover, only 20 subjects (12.0%) met DM criteria, according to the American Diabetes Association (ADA).

### Genotyping Frequency of *STRA6* SNPs in NSCLC Patients

Genotyping and allelic frequency are presented in **Table S2**. Four STRA6 SNPs (rs4886578, rs736118, rs351224, and rs974456) were analyzed, and all allelic frequencies were under Hardy-Weinberg assumption criteria, i.e., they are in genetic equilibrium (**Table S2**).

### Association Between the Clinical Characteristics and the *STRA6* SNPs

The relationship between clinical characteristics and *STRA6* polymorphisms was analyzed, and all findings are summarized in **Table 2**. Remarkable, TT genotype in SNP rs4886578 was correlated with non-smokers vs. current smokers (71.4 vs 28.6%, p = 0.038), and EGFR mutated vs wild-type (71.4 vs 28.6%, p = 0.001).

**Table 2:** Correlation among different polymorphisms and clinical characteristics in patients with NSCLC (N = 196).

	SNP rs4886578		p-Value	SNP rs736118		p-Value	SNP rs351224		p-Value	SNP rs974456		p-Value
	CC/CT	TT		CC/CT	TT		AA/AT	TT		CC/CT	TT	
	% (n/N)	% (n/N)		% (n/N)	% (n/N)		% (n/N)	% (n/N)		% (n/N)	% (n/N)	
**Gender**												
Female	54.9 (96/175)	66.7 (14/21)	0.303	54.8 (97/177)	68.4 (13/19)	0.256	57.6 (102/177)	42.1 (8/19)	0.195	56.4 (93/165)	54.8 (17/31)	0.875
Male	45.1 (79/175)	33.3 (7/21)		45.2 (80/177)	31.6 (6/19)		42.4 (75/177)	57.9 (11/19)		43.6 (72/165)	45.2 (14/31)	
**Age**												
≤60 yrs	46.9 (82/175)	33.3 (7/21)	0.240	48.0 (85/177)	21.1 (4/19)	**0.029**	44.6 (79/177)	52.6 (10/19)	0.506	47.3 (78/165)	35.5 (11/31)	0.226
>60 yrs	53.1 (93/175)	66.7 (14/21)		52.0 (92/177)	78.9 (15/19)		55.4 (98/177)	47.4 (9/19)		52.7 (87/165)	64.5 (20/31)	
**Tobacco exposure**												
Non-smoker	47.7 (83/175)	71.4 (15/21)	**0.038**	48.0 (85/177)	68.4 (13/19)	0.091	50.3 (89/177)	47.4 (9/19)	0.809	49.7 (82/165)	51.6 (16/31)	0.845
Smoker	52.6 (92/175)	28.6 (6/21)		52.0 (92/177)	31.6 (6/19)		49.7 (88/177)	52.6 (10/19)		50.3 (83/165)	48.4 (15/31)	
**Histology**												
Other	14.9 (26/175)	4.8 (1/21)	0.319	15.3 (27/177)	0 (0)	0.081	12.4 (22/177)	26.3 (5/19)	0.150	13.9 (23/165)	12.9 (4/27)	1.000
Adenocarcinoma	85.1 (149/175)	95.2 (20/21)		84.7 (150/177)	100.0(19/19)		87.6 (155/177)	73.7 (14/19)		86.1 (142/165)	87.1 (27/31)	
**EGFR mutation status**												
wt-EGFR	66.9 (117/175)	28.6 (6/21)	**0.001**	65.0 (115/177)	42.1 (8/19)	**0.050**	62.1 (110/177)	68.4 (13/19)	0.591	64.8 (107/165)	51.6 (16/31)	0.162
EGFR (+)	33.1 (58/175)	71.4 (15/21)		35.0 (62/177)	57.9 (11/19)		37.9 (67/177)	31.6 (6/19)		35.2 (58/165)	48.4 (15/31)	

EGFR (+), patients with mutations in EGFR.

Statistically significant values are in bold.

The homozygous SNP rs736118 (TT) showed an association with older (> 60 years) patients, (78.9% vs ≤ 60 years 21.1%, p = 0.029) and a positive *EGFR* mutational status vs non-mutated (57.9 vs 42.1%; p = 0.05) (**Table 2**). No differences existed regarding gender, age, ECOG PS, wood-smoke, or asbestos exposure with this polymorphism. None of the clinical variables were associated with the remaining *STRA6* polymorphisms.

### PFS and Clinical Features

**Figure 1:**
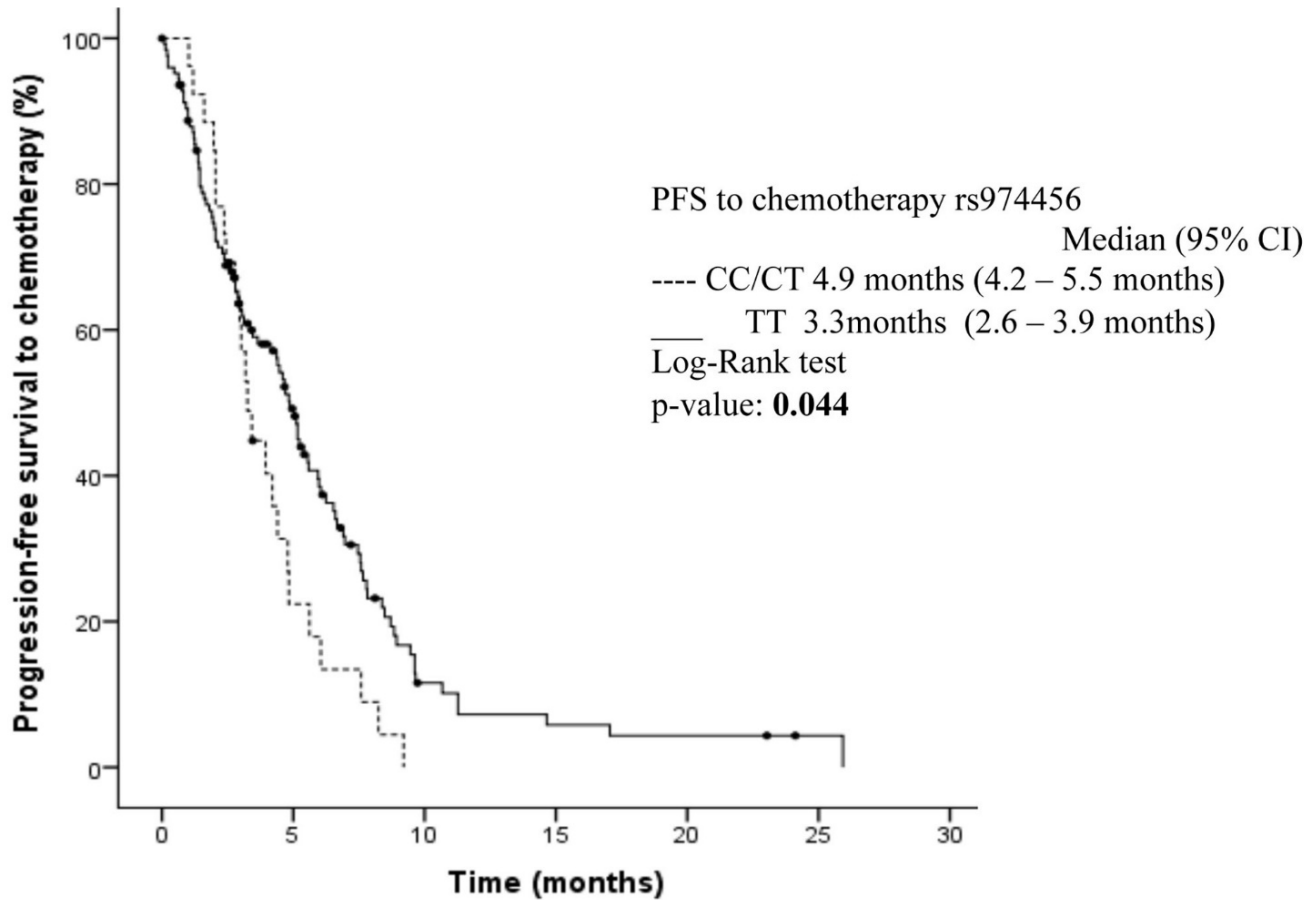
Kaplan–Meier curves for progression-free survival depending on rs974456 expression. Circles on the Kaplan–Meier curves represent censored observations.

The median PFS to platinum-based chemotherapy as first-line therapy was 4.6 months. Locally-advanced (stage III) vs. metastatic disease (stage IV) (5.5 vs. 4.3 months, *p* = 0.027) were associated with a better PFS. Additionally, patients with CC/CT genotype in the rs974456 polymorphism showed a better median PFS than those with a TT genotype (4.9 vs. 3.3 months, *p* = 0.044) (**Figure 1**). However, in the multivariate analysis, the clinical-stage was the only significant variable representing higher hazards of death [HR 1.85, 95% CI (1.0–3.3), p = 0.038] (**Table 3**).

**Table 3:** Univariate and multivariate analysis of the baseline factors associated with patient PFS and OS.

			Progression-Free Survival				Overall Survival					
			Univariate		Multivariate		Univariate			Multivariate		
			Median, (95% CI)	p-Value	HR, (95% CI)	p-value	Median, (95% CI)	p-Value		HR, (95% CI)	p-value	
**Overall**			4.6 (3.86–5.49)				34.9 (26–43.8)				
**Gender**											
	Female		4.8 (4.05–5.60)	0.651			38.0 (27.9–48.2)		0.732		
	Male		3.9 (2.25–5.63)				29.3 (20.4–38.2)				
**Age (yrs)**											
	< 60 yrs		4.6 (3.88–5.50)	0.723			43.9 (33.4–54.4)		**0.024**	1.18 (0.7–1.9)	0.502
	60+ yrs		4.1 (2.35–8.92)				26.9 (21.0–32.9)				
**Smoking History**											
	No		4.7 (4.15–5.44)	0.987			39.0 (28.9–49.1)		0.193		
	Yes		3.9 (2.23–5.65)				27.5 (19.4–35.7)				
**ECOG-PS**											
	0–1		4.6 (3.97–5.36)	0.065			39.0 (30.0–48.0)		**<0.001**	1.87 (1.15–3.1)	**0.011**
	2–3		2.7 (0.00–5.67)				13.3 (0.00–27.0)				
**Histology**											
	Adenocarcinoma		4.4 (3.30–5.50)	0.282			36.2 (27.3–45.2)		0.461		
	Other		5.5 (3.14–7.95)				24.2 (3.4–45.0)				
**Disease Stage**											
	III		5.5 (4.07–7.09)	**0.027**	1.85 (1.0–3.3)	**0.038**	76.9 (NR)		0.123	2.25 (1.2–3.1)	**0.035**
	IV		4.3 (3.20–5.53)				32.0 (23.3–40.3)				
**EGFR Mutation**											
	No		4.4 (3.09–5.70)	0.821			20.4 (13.4–27.5)		**<0.001**	2.73 (1.6–4.5)	**<0.001**
	Yes		4.6 (3.80–5.52)				47.2 (43.07–51.4)				
**Glycaemia**											
	< 120 mg/dL		4.1 (2.74–5.53)	0.728			38.9 (25.8–52.09)		**0.001**	1.52 (0.95–2.45)	0.079
	> 120 mg/dL		5.6 (3.34–7.88)				17.8 (6.8–28.7)				
**SNP rs4886578**											
	CC/CT		4.6 (3.79–5.53)	0.930			32.6 (23.9–41.3)		0.355		
	TT		3.2 (1.51–4.99)				40.9 (24.6–57.2)				
**SNP rs736118**											
	CC/CT		4.6 (3.92–5.41)	0.245			34.9 (25.7–44.1)		0.957		
	TT		3.02 (2.60–3.44)				32.0 (17.3–46.7)				
**SNP rs351224**											
	AA/AT		4.4 (3.44–5.36)	0.402			32.0 (24.2–39.8)		0.156	0.57 (0.2–1.3)	0.193
	TT		4.8 (1.76–7.95)				47.5 (15.4–79.5)				
**SNP rs974456**											
	CC/CT		4.9 (4.17–5.54)	**0.044**	1.54 (0.9–2.4)	0.063	36.2 (27.1–45.5)		0.824		
	TT		3.3 (2.62–3.88)				27.1 (15.3–38.9)				

SD, standard deviation; ECOG, European Cooperative Eastern Group Classification.

Statistically significant values are in bold.

### OS and Clinical Features

The median overall survival (OS) in the whole population was 34.9 months. Concerning the presence of STRA6 SNPs, patients with a TT genotype in rs351224 showed a longer median OS (47.5 vs. 32.0 months; p = 0.156) compared with those without this polymorphism; however, this difference was not statistically significant. In the univariate analysis, age ≤60 years vs >60 years (43.9 vs 26.9 months, *p* = 0.024); ECOG PS (0–1) vs (≥ 2) (39 vs 13.3 months, *p* = < 0.001), positive *EGFR* mutation (47.2 vs 20.4 months, p < 0.001); and glycaemia <120 mg/dL (38.9 vs 17.8 months, *p* = 0.001) were associated to an improvement in OS. No other characteristics were significant for OS (**Table 3**). In the multivariate analysis, just early-stage disease, ECOG PS (0–1), and *EGFR* mutated status were significant for an improvement in OS (**Table 3**).

The TT homozygous condition in SNP rs4886578 was associated with a non-smoker history [0.406 (0.142–1.169), p = 0.042; HR, 95%CI] and a positive EGFR mutation [0.205 (0.073–0.575), p = 0.003; HR, 95%CI]; likewise, TT homozygous condition in SNP rs736118 was associated to a positive EGFR mutation [4.773 (1.462–15.583), p = 0.010; HR, 95%CI] (**Table S3**).

### SNP Validation in NSCLC Samples by Sequencing

Following the screening analysis for *STRA6* allelic discrimination, the detection of polymorphisms was validated by mean Sanger sequencing; notably, results showed 100% concordance with those previously obtained by RT-PCR.

### Linkage Disequilibrium and Haplotype Analysis of *STRA6* SNP’s

**Figure 2:**
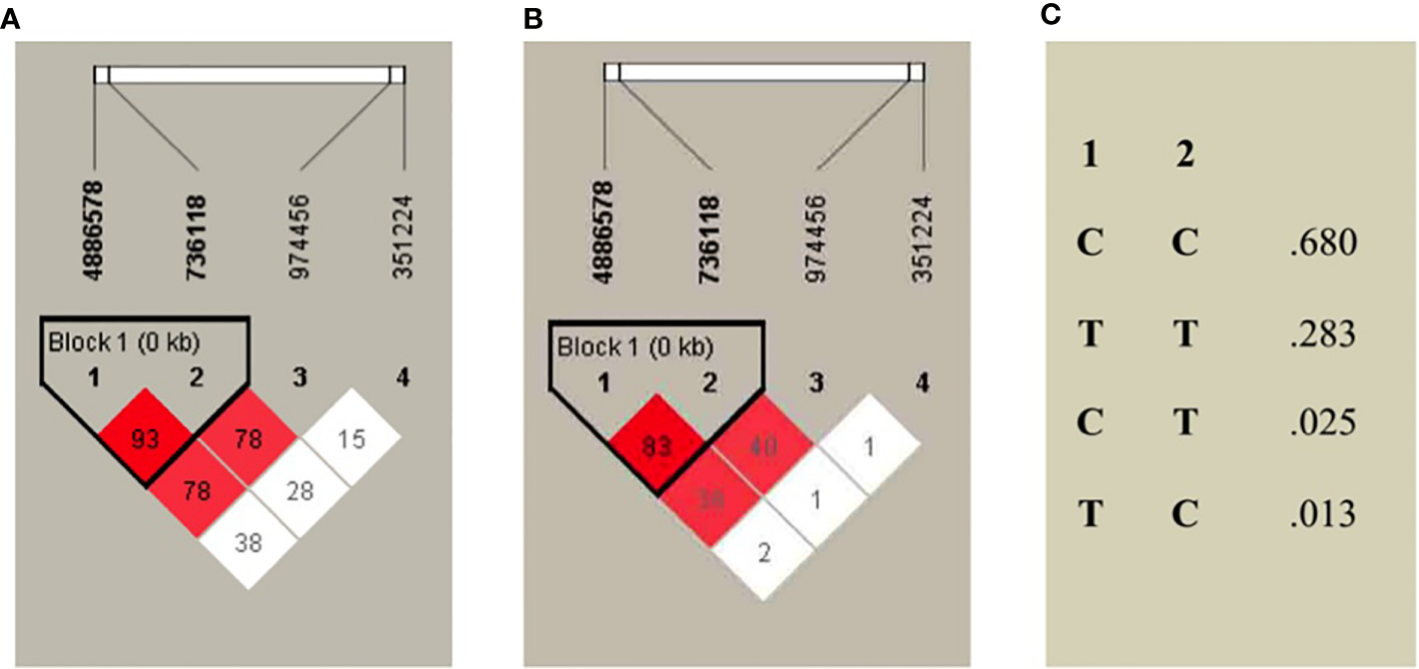
Linkage Disequilibrium Diagram of four STRA6 SNPs in NSCLC patients. **(A)** Shows D’ values and **(B)** r2 values, indicated in dark red when linkage disequilibrium is solid (LOD ≥ 2, D’ = 1); in light red for intermediate DL (LOD ≥ 2, D’ < 1) and in white when there is no LD (LOD < 2, D’ < 1). **(C)** Haplotype frequency in *STRA6* (1: rs4886578; 2: rs736118). LOD, Likelihood of Odds.

Linkage disequilibrium (LD) between loci couples was performed based on RT-PCR genotyping results, using Haploview software. Haplotype presence was studied in the population-based on Gabriel’s definition (D’ > 0.9; minimal allelic frequency >5%) (29). We found three red points of LD between SNPs (one intense and two weaker). The four S*TRA6* SNPs are shown in **Figures 2A, B**, with a color code (dark red = strong LD; light red = intermediate LD and white = in equilibrium). According to our data, there is only one block of LD in STRA6 among the analyzed population. The SNP rs351224 variant was in equilibrium concerning other variants (rs4886578, rs736118, and rs974456). There is a strong LD between rs4886578 and rs736118, followed by intermediate LD between rs4886578, rs736118, and rs974456 (**Figures 2A, B**).

The analysis of all data performed on the study population investigates the possible haplotype and the value of confidence interval pre-established by Gabriel et al. using an *r^2^ = 0.8* (29). Concerning the haplotype (rs4886578/rs736118), 68.0% showed CC, 28.3% TT, and 2.5% CT, respectively (**Figures 2A, C**).

### Clinical Features of Haplotype (TT/TT, rs4886578/rs736118)

An analysis of whether the presence of a specific haplotype was associated with clinical features was performed. **Table 4** summarizes the main findings. The TT/TT haplotype (rs4886578/rs736118) was correlated with the presence of an *EGFR* mutation (64.7 vs. 35.3%, p = 0.019) compared with wild-type patients. No other clinical characteristics showed any association with this haplotype. Additionally, 8.1% (16/196) of the patients with a TT genotype for SNP rs4886578 also have the TT genotype for rs351224 and rs974456. TT genotype was associated with older patients (> 60 years), 81.3 vs 18.8%, *p* = 0.034 and EGFR mutated status, 62.5 vs 37.5%, *p* = 0.029 (**Table 4**).

**Table 4:** Correlation among different haplotypes and clinical characteristics in patients with NSCLC (N = 196).

	rs4886578/rs736118		p-Value	rs4886578/rs351224/rs974456		p-Value
	CC-CT/CC-CT	TT/TT		CC-CT/AA-AT	TT/TT	
	% (n/N)	% (n/N)		% (n/N)	% (n/N)	
**Gender**						
Female	54.7 (98/179)	70.6 (12/17)	0.307	55.0 (99/180)	68.8 (11/16)	0.431
Male	45.3 (81/179)	29.4 (5/17)		45.0 (81/180)	31.3 (5/16)	
**Age**						
≤ 60 yrs	47.5 (85/179)	23.5 (4/17)	0.075	47.8 (86/180)	18.8 (3/16)	**0.034**
> 60 yrs	52.5 (94/179)	76.5 (13/17)		52.2 (94/180)	81.3 (13/16)	
**Tobacco exposure**						
Non-smoker	48.0 (86/179)	70.6 (12/17)	0.126	48.3 (87/180)	68.8 (11/16)	0.191
Smoker	52.0 (93/179)	29.4 (5/17)		51.7 (93/180)	31.3 (5/16)	
**Histology**						
Other	15.1 (27/179)	0 (0)	0.136	15.0 (27/180)	0 (0)	0.135
Adenocarcinoma	84.9 (152/179)	100.0 (17/17)		85.0 (153/180)	100.0 (16/16)	
**EGFR mutation status**						
wt-EGFR	65.4 (117/179)	35.3 (6/17)	**0.019**	65.0 (117/180)	37.5 (6/16)	**0.029**
EGFR (+)	34.6 (62/179)	64.7 (11/17)		35.0 (63/180)	62.5 (10/16)	

EGFR (+), patients with mutations in EGFR.

Statistically significant values are in bold.

Significantly, a homozygous condition in both haplotypes was associated with older age (> 60 years) and EGFR mutated status (**Table 4**). Nevertheless, no association of these haplotypes with PFS or OS was observed (**Table S4**).

## Discussion

Currently, STRA6 is the only known retinol transporter mediating its entry into the cell cytoplasm. The association of the RBP-retinol complex with STRA6 acts as a cytokine downstream activation receptor in the JAK2-STAT signaling pathway (30, 31). Previously, STRA6 was overexpressed in breast and colon tumor tissue compared with healthy tissue (32); however, it is unknown the expression or variation within lung cancer.

In the present study, we analyzed four STRA6 SNPs in patients with a lung cancer diagnosis, and all were in Hardy-Weinberg equilibrium. According to The Genome Aggregation Database, (gnomAD, https://gnomad.broadinstitute.org/), all four SNPs studied were present in Latin-American populations. However, no previous studies were conducted in our population; thereby, STRA6 function or mutations are not well defined in Mexican patients. We demonstrated that homozygous TT genotypes in STRA6 SNPs rs4886578 and rs736118 were associated with essential and prognostic clinical characteristics, such as age (≥ 60 years), non-smokers, and EGFR mutated status. By screening the public database VarSome (https://varsome.com), three of four SNPs analyzed in our study occurred in a small proportion of cancer samples; however, none of them in lung cancer specimens. The SNP rs4886578 was found only in 2 of 402 liver cancer samples; SNP rs736118 in 4 of 321 colorectal cancer samples, in 1 of 205 liver cancer and 1 of 402 acute myeloid leukemia samples. The SNP rs351224 was present in only 1 of 205 acute myeloid leukemia samples (VARSOME) (33). Unfortunately, none of the SNP have been associated with clinical features; moreover, according to the database, all previously mentioned SNPs have a benign role; thereby, more robust evidence is in need to define SNPs’ role in lung cancer pathogenesis.

On the other hand, the association between STRA6 polymorphisms and type 2 diabetes mellitus (DM2) has been studied in Indian and Chinese populations. In both studies, the same STRA6 SNPs were analyzed, like in the present work. In the Indian population, there was a positive correlation between haplotype (AAT-rs974456/rs736118/rs4886578) and the presence of DM2 (26). Likewise, in the Chinese population, the rs736118 and rs974456 SNPs were associated with DM2 (27). In Mexico, approximately 10% of the population has DM2. Our group showed that NSCLC patients with DM2 but an adequate glycemic index had better survival than hyperglycemic states (34). In the present study, 15% of the patients had a DM2 confirmed diagnosis. Although it was not part of the main objectives, we analyzed the possible association between the expression of the STRA6 SNPs with DM2, and in contrast with previous studies, we did not found connections between both conditions. However, according to our previous findings, patients without DM2 diagnosis and lower blood glucose levels than 120 mg had better median PFS.

TT genotype SNP rs4886578 and rs736118 were associated with the presence of an EGFR mutated status. A larger proportion of Latin American patients with NSCLC harbor EGFR mutations than other races, even more, in non-smokers, young women, and history of wood-smoke exposure; similar findings in the current study have been described (35–37). Two of the SNPs (rs4886578 and rs736118) might probably serve as potential biomarkers in non-smokers harboring EGFR mutations, although the relation between the EGFR pathway and STRA6 function remains unknown. We made an additional linear statistical analysis, strengthening the relationship between the TT haplotype of both SNPs and the EGFR mutations presence (**Table S4**). EGFR mutations can induce sustained downstream signaling activation to induce proliferation, differentiation, and survival. At the same time, EGFR signaling could activate the STAT3 (signal transducer and activator of transcription 3) pathway (38), which can stimulate *via* STRA6 activation. Although the other two SNPs do not show associated clinical characteristics, the TT genotype of rs974456 showed a statistical association with shorter PFS, suggesting a role as a poor prognosis biomarker in NSCLC patients.

The rs736118 SNP is a G>A polymorphism that leads to a Met to Ile substitution in the C-terminal of STRA6. According to the literature, this change can alter protein trafficking and cell surface expression (26). Even though the other three SNPs are localized in gene intron regions, SNPs in non-coding regions can modulate gene expression (39). Since STRA6 modulates retinol entry to the cytoplasm, the TT genotype of rs736118 or rs974456 could modify the cellular membrane’s STRA6 function. Thus, limiting the retinol intake and inhibiting nuclear receptors’ expression and activity leads to shorter PFS and OS, supporting its probable significance as a poor prognosis predictor. Previously we have reported that loss of RARα and RARβ expression in tumor tissue from advanced NSCLC patients was associated with a worse prognosis (40). Indeed, in an independent study, advanced NSCLC patients who received chemotherapy complemented with all-trans-retinoic acid showed a better overall response rate than those who received chemotherapy alone (55.8 vs. 25.4%, respectively). Moreover, a small patient subgroup who expressed RARβ on tumor tissue showed a better response (66.6%) than those without RARβ expression (42%) (9).

In contrast, it seems that the TT genotype SNP rs351224 might not influence STRA6 localization or function, but could induce an overactivation, facilitating the retinol entry to the tumor cell, high expression of nuclear receptors, and favorable PFS and OS outcomes. Despite the survival benefit was not significant for patients with a TT genotype SNP rs351224, although a strong trend was observed. Patients with the TT genotype rs351224 did not exhibit a concomitant presence of any other SNP; thus, it could be considered an independent factor compared to other SNPs. An intriguing topic would be to define the role of the TT genotype SNP rs351224 in NSCLC patients under retinoic acid substitution in combination with systemic therapy, and whether it could be adopted as a prognosis biomarker.

Two STRA6 polymorphisms have been related as a haplotype (rs4886578/rs736118) with prognostic clinical characteristics. First, this haplotype was correlated to an EGFR mutated status supporting the rationale that both SNPs by itself could participate in the development of lung cancer with non-smoking history. Although the statistical analysis was not significant, it was possible to observe a slightly trending of a more prolonged OS in those patients with TT/TT genotype in the haplotype (rs4886578/rs736118). This tendency disappeared when the patients were homozygous for three SNPs (rs4886578/rs736118/rs974456); it reinforces the assumption that rs974456 TT genotype might be an adverse prognostic marker.

We propose STRA6 expression could be related to normal retinol levels in the tumor cell cytoplasm, favoring expression of retinol nuclear receptors, and playing an essential role in NSCLC patients as improving oncological outcomes. Nuclear receptors expression depends on the retinoic acid concentration in the cellular cytoplasm. STRA6 function could be essential for the downstream activation pathway, strengthening the hypothesis as a determinant role in lung carcinogenesis. Further research regarding the STRA6 expression in tumor tissue and its relation with RARs expression and clinical outcome in advanced NSCLC patients is in process.

Our study had some limitations due to the small and heterogeneous sample size. Moreover, important variations in allelic frequencies between races have been described, thus, it will be attractive to explore STRA6 SNPs in other populations. Ideally should be pursued in larger cohorts of patients to increase validity in subgroups analyses. To the best of our knowledge, this is the first study that addresses a potential association between *STRA6* SNPs, relevant clinical characteristics, and oncological outcomes in NSCLC patients and attractive results have emerged in this first analysis. However, it remains to confirm if SNPs rs4886578 and rs736118 participate in lung cancerogenesis regardless of tobacco exposure, through stratifying patients based on EGFR mutational status. There is an urgent need to develop reliable biomarkers; in this context, it is relevant to discern whether any STRA6 SNPs could help to establish prognosis or predict benefits with current standards of treatment.

## Conclusion

A positive association between the TT genotype SNP rs4886578 and rs736118, individually and in the haplotype form, was observed with non-smoking history and EGFR mutational status; suggesting its involvement in the genesis of lung cancer unrelated to tobacco exposure.

## Data Availability Statement

The raw data supporting the conclusions of this article will be made available by the authors, without undue reservation.

## Ethics Statement

The studies involving human participants were reviewed and approved by Bioethics and scientific INCan (Instituto Nacional de Cancerología Mexico) committees (015/026/IBI) (CEI/954/15). The patients/participants provided their written informed consent to participate in this study. 

## Author Contributions

Conceptualization: SM-H, OA. Data curation: SM-H, OA. Formal analysis: SM-H, ML-L, OA. Funding acquisition: SM-H, OA. Investigation: SM-H, OM-F, YM-L, and AO. Methodology: SM-H, JV-F, and JD-C. Project administration: SM-H. Resources: SM-H, ML-L, OA. Supervision: SM-H, ML-L, OA. Writing-Original draft: SM-H, OA. Writing-review and editing: SM-H, JV-F, JD-C, OM-F, YM-L, AO, ML-L, and OA. All authors contributed to the article and approved the submitted version.

## Funding

This work was partially financed by the Instituto Nacional de Cancerología of Mexico. 

## Conflict of Interest

The authors declare that the research was conducted in the absence of any commercial or financial relationships that could be construed as a potential conflict of interest. 
