# Correction: STRA6 polymorphisms are associated with EGFR mutations in locally-advanced and metastatic non-small cell lung cancer patients

**DOI:** 10.3389/fonc.2025.1760931

**Published:** 2025-12-19

**Authors:** Saé Muñiz-Hernández, Jesús Bernardino Velázquez-Fernández, José Díaz-Chávez, Omar Mondragón-Fonseca, Yerye Mayén-Lobo, Alberto Ortega, Marisol López-López, Oscar Arrieta

**Affiliations:** 1 Laboratorio de Oncología Experimental, Subdirección de Investigación Básica, Instituto Nacional de Cancerología, Ciudad de México, Mexico; 2 Unidad de Tecnología Ambiental, Centro de Investigación y Asistencia en Tecnología del Estado de Jalisco, Jalisco, Mexico; 3 Laboratorio de Carcinogénesis, Dirección de Investigación, Instituto Nacional de Cancerología, Ciudad de México, Mexico; 4 Laboratorio de Genética Molecular, Departamento de Sistemas Biológicos, Universidad Autónoma Metropolitana-Xochimilco, Ciudad de México, Mexico; 5 Unidad de Oncología Torácica, Instituto Nacional de Cancerología, Ciudad de México, Mexico

**Keywords:** non-small cell lung cancer, single nucleotide polymorphisms, stimulated by retinoic acid 6 (STRA6), genotype, retinol pathways

In the published article, the legend of **Figure 1** did not specify the meaning of the circles on the Kaplan–Meier curves.

The caption for **Figure 1** has been updated to read:

“Kaplan–Meier curves for progression-free survival depending on rs974456 expression. Circles on the Kaplan–Meier curves represent censored observations.”

**Figure 1:**
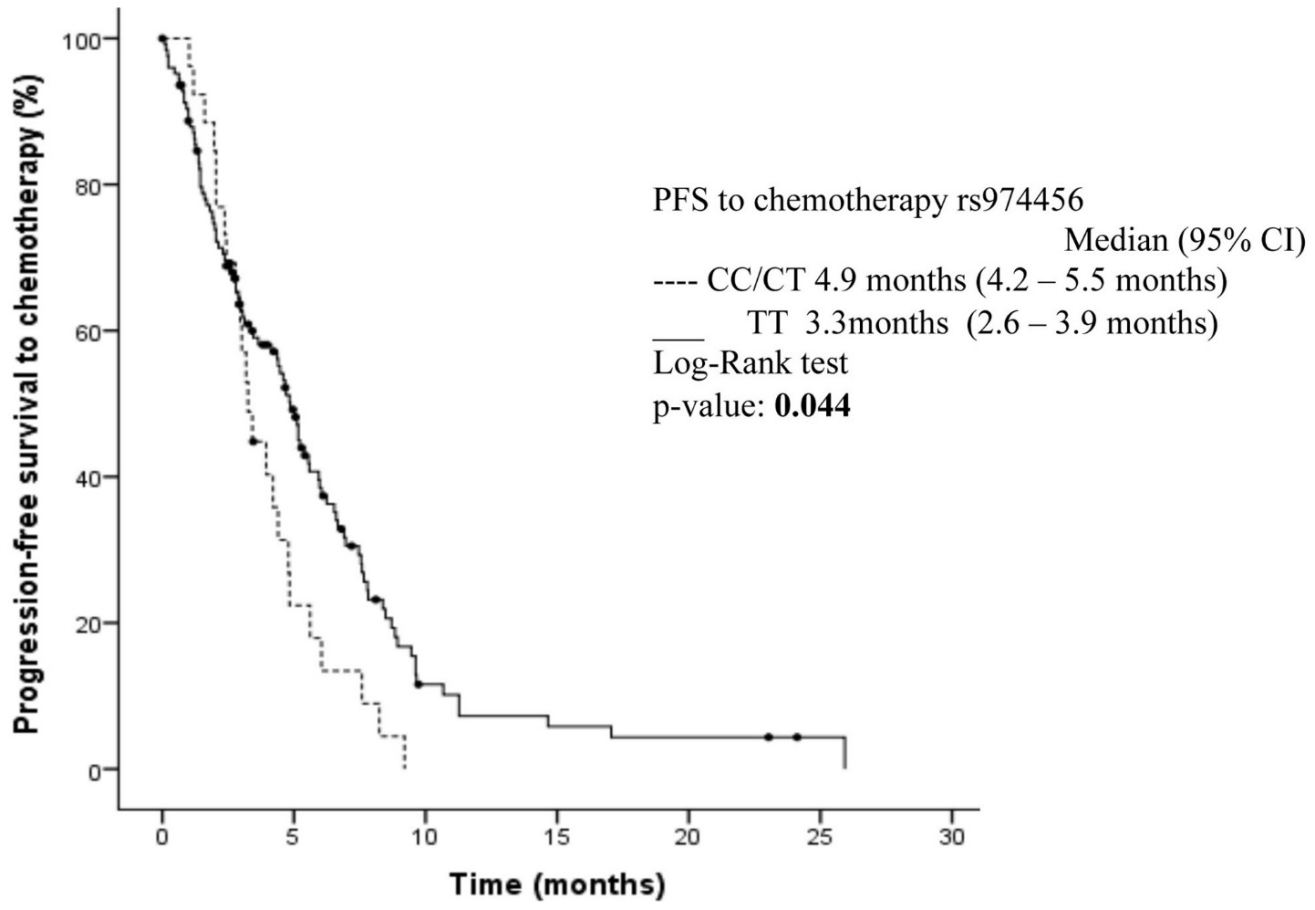
Kaplan–Meier curves for progression-free survival depending on rs974456 expression. Circles on the Kaplan–Meier curves represent censored observations.

In the published article, the definition of progression-free survival (PFS) in the *Statistical Analysis* section was imprecise and could be interpreted as treating unacceptable toxicity and loss to follow-up as PFS events. In all survival analyses, however, PFS events consisted only of radiologic or clinical disease progression or death from any cause; patients who discontinued treatment due to unacceptable toxicity or were lost to follow-up were censored at the date of last disease assessment or last contact.

A correction has been made to the section *Statistical Analysis*, where the PFS definition previously read:

“PFS was defined as the time to an event from the date of initiating treatment until disease progression, unacceptable toxicity, death, or loss to follow-up.”

The corrected text now reads:

“Progression-free survival (PFS) was defined as the time from the date of initiating treatment to radiologic or clinical disease progression or death from any cause, whichever occurred first. Patients without progression or death, including those who discontinued treatment due to unacceptable toxicity or were lost to follow-up, were censored at the date of last disease assessment or last contact.”

The original version of this article has been updated.

