# Supplementary material for: STRA6 Polymorphisms Are Associated With EGFR Mutations in Locally-Advanced and Metastatic Non-Small Cell Lung Cancer Patients
Source: Front Oncol. 2020 Nov 24;10:579561. doi: 10.3389/fonc.2020.579561 (PMC7723324; doi:10.3389/fonc.2020.579561)
Supplement: Supplementary file 1 [file Table_1.docx]

Supplementary Material

# Supplementary Tables

| **Table S1. Treatment regimen in the study population** | | |
| --- | --- | --- |
|  |  | N=196  % (n/N) |
| **Chemotherapy platinum-regimen** |  |  |
| CDP/paclitaxel |  | 17.9 (35/196) |
| CBP/paclitaxel |  | 47 (92/196) |
| CBP/GMZ |  | 7.1 (14/196) |
| Other |  | 12.2 (24/196) |
| No chemotherapy |  | 15.8 (31/196) |
| **TKIs** |  |  |
| Afatinib |  | 16.8 (33/196) |
| Erlotinib |  | 7.7 (15/196) |
| Gefitinib |  | 17.9 (35/196) |
| Other |  | 1.5 (3/196) |
| No TKIs |  | 56.1 (110/196) |
| CDP, cisplatin; CBP, carboplatin; GMZ, gemcitabine; TKIs, Tyrosine Kinase Inhibitors | | |

| **Table S2. Genotyping and Allelic frequency of STRA6 SNPs** | | | | | | |
| --- | --- | --- | --- | --- | --- | --- |
| Genetic Variant  *(GRCh38.p12 Chr 15)* | | **Genotype** | **n** | **Genotype**  **Frequency** | **Allele** | **Allelic**  **Frequency** |
| g.74180694C>T  (rs4886578) | | CC | 95 | 0.485 (0.413-0.557) | C | 0.699 |
|  |  | CT | 84 | 0.429 (0.358-0.501) |  |  |
|  |  | TT | 17 | 0.087 (0.051-0.135) | T | 0.301 |
| g. 74181398C>T  (rs736118) | | CC | 90 | 0.459 (0.388-0.532) | C | 0.681 |
|  |  | CT | 87 | 0.444 (0.373-0.516) |  |  |
|  |  | TT | 19 | 0.097 (0.059-0.147) | T | 0.319 |
| g.74194695T>A  (rs351224) | | AA | 100 | 0.510 (0.438-0.582) | A | 0.712 |
|  |  | AT | 79 | 0.403 (0.334-0.475) |  |  |
|  |  | TT | 17 | 0.087 (0.051-0.135) | T | 0.288 |
| g.74194103C>T  (rs974456) | | CC | 65 | 0.332 (0.266-0.402) | C | 0.592 |
|  |  | CT | 102 | 0.520 (0.448-0.592) |  |  |
|  |  | TT | 29 | 0.141 (0.101-0.205) | T | 0.408 |
|  |  |  |  |  |  |  |

| **Table S3. Factors related to STRA6 SNPs** | | | | | | | | | |  |  |
| --- | --- | --- | --- | --- | --- | --- | --- | --- | --- | --- | --- |
|  |  | **rs4886578** | | |  | | **rs736118** | | |  |  |
|  | **HR,**  **(95% CI)** | | **p-value** |  | | **HR,**  **(95% CI)** | | | **p-value** | | |
| **Age 60+yrs** | 2.593  0.939 – 7.162 | | 0.66 |  | | 0.388  0.141 – 1.072 | | 0.068 | | |  |
| **Non-smoker** | **0.406**  **0.142 – 1.160** | | **0.042** |  | | 0.391  0.134 – 1.140 | | 0.085 | | |  |
| **EGFR-mutations** | **0.205**  **0.073 – 0.575** | | **0.003** |  | | **4.773**  **1.462 – 15.582** | | **0.010** | | |  |
|  | | | | | | | | | |  |  |

| **Table S4. Univariate analysis of haplotypes associated with PFS and OS of patients** | | | | | | |  |
| --- | --- | --- | --- | --- | --- | --- | --- |
| **Haplotype** |  | **PFS** | |  | **OS** | | |
|  |  | Median, 95%CI | p-Value |  | Median, 95%CI | p-Value | |
| **rs4886578/rs736118** |  |  |  |  |  |  | |
| **CC-CT/CC-CT** |  | 4.7 (3.9 - 5.3) | 0.499 |  | 34.9 (25.7 - 44.2) | 0.557 | |
| **TT/TT** |  | 3.2 (2.8 - 3.6) |  |  | 40.9 (23.9 - 57.9) |  | |
| **rs4886578/rs736118/**  **rs974456** |  |  |  |  |  |  | |
| **CC-CT/CC-CT/CC-CT** |  | 4.7 (3.9 - 5.4) | 0.307 |  | 34.9 (25.7 - 44.1) | 0.724 | |
| **TT/TT/TT** |  | 3.2 (2.9 - 3.5) |  |  | 32.0 (16.3 - 47.8) |  | |
